# Bendamustine and Rituximab Treatment, Chronic Lymphocytic Leukemia, Direct Antiglobulin Test, and False Negatives

**DOI:** 10.4274/tjh.galenos.2018.2018.0397

**Published:** 2019-02-07

**Authors:** Won Sriwijitalai, Viroj Wiwanitkit

**Affiliations:** 1 TWS Medical Center, Bangkok, Thailand; 2 Dr. DY Patil University, Pune, India

**Keywords:** Bendamustine, Rituximab, Leukemia, Direct antiglobulin test, False negatives

## To the Editor,

We read “Early Direct Antiglobulin Test Negativity After Bendamustine and Rituximab Treatment in Chronic Lymphocytic Leukemia: Two Cases” [1]. Eren and Suyanı noted that “BR seems to be an important treatment of choice in terms of eliminating the poor prognostic factor of direct antiglobulin test (DAT) positivity and assuring safe cessation of steroid treatment due to rapid achievement of DAT negativity” [1]. The interesting observation of a negative DAT test should be discussed. There is another possibility that Eren and Suyanı did not mention. In their report, Eren and Suyanı noted that no titer was provided [1]. Whether the negative result is a false negative result should be discussed. For DAT testing, the prozone phenomenon is observable and it is important to consider this phenomenon in the interpretation of unexpected false negative DAT tests [2,3,4].
