# Supplementary material for: Bendamustine and Rituximab Treatment, Chronic Lymphocytic Leukemia, Direct Antiglobulin Test, and False Negatives
Source: Turk J Haematol. 2019 Feb 7;36(1):53–4. doi: 10.4274/tjh.galenos.2018.2018.0397 (PMC6373499; doi:10.4274/tjh.galenos.2018.2018.0397)
Supplement: Related texts

# Reply to the Authors

**Authors:** Rafet Eren, Elif Suyanı

## Dear Editor,

In their comments, Won Sriwijitalai and Viroj Wiwanitkit pointed out that the possibility of false negative direct antiglobulin test (DAT) was not denoted in our paper in which we shared our experience about the achievement of early DAT negativity in patients receiving bendamustine and rituximab treatment for chronic lymphocytic leukemia [1,2]. Along with the prozone phenomenon that the authors mentioned, there are other causes of a false negative DAT, including poor washing technique, improper agitation of specimen during reaction strength determination, failure to add or delayed addition of antihuman globulin reagent, inactive antihuman globulin reagent, inappropriately concentrated red blood cell suspension, and delay in testing [3]. However, we used an automated gel centrifugation method sytem to detect DAT which ensured quite accurate results. Also, the reported patients had normal hemoglobin, haptoglobulin, LDH, and indirect bilirubin levels during DAT negativity, suggesting that the existence of a high titer of antibody, the reason of prozone affect, was unfeasible. Additionally, sustained DAT negativity during follow-up led to the consideration that DAT negativity was accurate and false negative DAT was unlikely. 

**Best Regards,**

## References

[1] Eren R, Suyanı E (2018). Early direct antiglobulin test negativity after bendamustine and rituximab treatment in chronic lymphocytic leukemia: two cases. Turk J Hematol.

[2] Sriwijitalai W, Wiwanitkit V (2019). Bendamustine and rituximab treatment, chronic lymphocytic leukemia, direct antiglobulin test, and false negatives. Turk J Hematol.

[3] Zantek ND, Koepsell SA, Tharp DR Jr, Cohn CS (2012). The direct antiglobulin test: a critical step in the evaluation of hemolysis. Am J Hematol.
